# Effectiveness of preimplantation genetic testing for aneuploidy in improving clinical and neonatal outcomes for patients with recurrent pregnancy loss

**DOI:** 10.3389/fendo.2025.1690361

**Published:** 2025-12-09

**Authors:** Jieliang Li, Zhiming Li, Xiaoli Lin, Zhanhui Ou, Junye Huo, Xiaowu Fang, Jing Du, Xiufeng Lin, Xiaojun Wen

**Affiliations:** Reproductive Medicine Center, Boai Hospital of Zhongshan, Zhongshan, Guangdong, China

**Keywords:** preimplantation genetic testing for aneuploidy, recurrent pregnancy loss, advanced maternal age, chromosomal polymorphism, ovarian reserve

## Abstract

**Objectives:**

This study aimed to evaluate a clinical utility of preimplantation genetic testing for aneuploidy (PGT-A) in patients with recurrent pregnancy loss (RPL) and explore a population that benefits from it.

**Methods:**

We analyzed 818 oocyte retrieval cycles and 1,165 frozen embryo transfer cycles from 797 patients with RPL. Stratified analyses were conducted based on age, number of miscarriages, chromosomal polymorphisms and ovarian reserve to compare clinical and perinatal outcomes between PGT-A and non-PGT-A groups.

**Results:**

Euploid, aneuploid, and mosaic blastocysts accounted for 54.07%, 33.33%, and 12.60%, respectively. Age stratification analysis showed that PGT-A significantly improved the clinical pregnancy rate (CPR) and live birth rate (LBR) in patients under 38 years (CPR: 63.64% vs. 55.03%, P = 0.004; LBR: 53.03% vs. 43.25%, P = 0.002). In age group of 38 years or older, PGT-A was associated with a higher CPR (50.63% vs. 35.42%, P = 0.034), while the increase in LBR did not reach statistical significance (36.70% vs. 23.96%, P = 0.084). In patients with three previous miscarriages, PGT-A significantly improved CPR (74.70% vs. 42.13%) and LBR (55.42% vs. 32.02%) (P < 0.05). Chromosomal polymorphism analysis revealed that outcomes with PGT-A were superior for patients with normal karyotypes, whereas no significant differences were observed among those with polymorphisms. PGT-A significantly improved CPR (59.68% vs. 52.37%, P = 0.004) and LBR (49.41% vs. 40.56%, P < 0.01) in patients with normal ovarian reserve. Although CPR increased in those with a diminished ovarian reserve (62.50% vs. 39.45%, P = 0.015), this was accompanied by a marked increase in the miscarriage rate (40.00% vs. 23.36%, P = 0.029). Consequently, no statistical difference was noted in LBR.

**Conclusions:**

PGT-A can significantly improve CPR and LBR in patients with RPL aged < 38 years with a history of three miscarriages, a normal chromosomal karyotype, and a normal ovarian reserve. For patients with a history of two miscarriages, PGT-A notably enhances a success of clinical pregnancy conversion into live birth. However, for individuals with diminished ovarian reserve, chromosomal polymorphisms, or a history of four or more miscarriages, a cautious and individualized assessment is necessary.

## 1 Introduction

*In vitro* fertilization and embryo transfer (IVF-ET) and intracytoplasmic sperm injection (ICSI) are key techniques for managing infertility. According to the American Society for Reproductive Medicine Practice Committee, recurrent pregnancy loss (RPL) is defined as the clinical loss of two or more consecutive pregnancies (1). Although the overall incidence of RPL among reproductive-aged couples is only 1–5% (2), it remains a significant clinical challenge in assisted reproductive technology (3–5). Etiological factors of RPL include genetic disorders, endocrine abnormalities, uterine anatomical defects, immune dysregulation, thrombophilic conditions, and infections. Among these, embryonic chromosomal abnormalities are considered the primary cause of early pregnancy loss (6), with 50–70% of affected embryos exhibiting aneuploidy (7).

Preimplantation genetic testing for aneuploidy (PGT-A) offers a method for screening chromosomal abnormalities. This technology has advanced from early fluorescence *in situ* hybridization, which could detect only a limited number of chromosomes, to current comprehensive chromosomal screening achieved through next-generation sequencing and array comparative genomic hybridization. To improve pregnancy outcomes and reduce miscarriage risk, existing guidelines recommend using PGT-A to select euploid embryos in patients with RPL (8–10). However, this technique may cause irreversible damage to embryos during the biopsy process, and there is a risk of discarding high-quality embryos, raising concerns about its clinical benefits. Several studies present conflicting evidence of PGT-A efficacy. For example, Vissenberg et al. reported that evidence supporting PGT-A for improving live birth rates in patients with RPL is currently insufficient (11), a conclusion supported by Murugappan et al. and Sato et al., who reported that PGT-A does not improve outcomes for patients with RPL (12, 13). Conversely, other studies have confirmed that PGT-A can increase clinical pregnancy rates, shorten time to conception, and alleviate the psychological and physical burden on patients (13, 14).

Most existing research conducts stratified analyses primarily by maternal age. Adamyan et al. reported significant differences in clinical outcomes between the PGT-A and conventional IVF/ICSI groups in patients with RPL aged ≥35 years, a disparity that was not observed in younger patients (15). Similarly, Liu et al. found that, even with PGT-A treatment, younger patients still exhibited a high miscarriage rate (6). However, few studies have stratified patients with RPL by other clinical characteristics, such as a history of multiple miscarriages, diminished ovarian reserve, or chromosomal polymorphisms in one or both partners, making it challenging to individualize PGT-A decision-making. Preliminary evidence suggests that pregnancy outcomes following the transfer of euploid embryos may vary depending on the number of previous miscarriages (16–18).

Given the potential value of PGT-A in RPL management and the existing controversies, this study aimed to systematically evaluate the clinical application of PGT-A in patients with RPL. We conducted stratified analysis by age, number of miscarriages, chromosomal polymorphisms, and ovarian function status to identify the subgroups most likely to benefit from PGT-A, thus providing evidence-based support for clinical decision-making.

## 2 Materials and methods

### 2.1 Study design and ethics approval

We conducted a retrospective analysis of 797 couples experiencing unexplained recurrent miscarriages who visited Boai Hospital in Zhongshan between May 2019 and September 2024, encompassing 818 oocyte retrieval cycles. The inclusion criteria were two or more recurrent miscarriages and implementation of frozen-thawed single blastocyst transfer. The exclusion criteria were as follows: 1) chromosomal karyotype abnormalities in either partner; 2) single-gene hereditary disease in either partner; 3) uterine anatomical abnormalities or endometriosis; 4) endocrine disorders or autoimmune diseases; 5) oocyte donation cycles; and 6) cycles without embryo transfer. In this study, some patients with RPL (PGT-A group) chose to undergo PGT-A testing to further exclude aneuploidy of embryos, which is a common cause of miscarriage, while other RPL patients chose not to undergo PGT-A due economic or other reasons. Based on whether PGT-A was performed, the oocyte retrieval cycles were divided into two groups: the PGT-A group (203 cycles for 200 couples) and the non-PGT-A group (615 cycles for 597couples). The PGT-A group underwent aneuploidy screening, while the non-PGT-A group received only IVF/ICSI. This study was approved by the Independent Ethics Committee of Boai Hospital, Zhongshan.

This study comprised the following two main components: first, an analysis and comparison of the embryonic chromosomal results in the PGT-A group of patients with RPL; and second, a stratified analysis based on the female patient’s age, number of miscarriages, chromosomal polymorphisms in either partner, and ovarian reserve, comparing clinical and perinatal outcomes between the PGT-A and non-PGT-A groups. Here, diminished ovarian reserve was defined as FSH > 10 IU/ml on day 3 of the menstrual cycle and/or AMH < 1 ng/ml (19).

### 2.2 Clinical treatment protocols, embryo culture, and biopsy

All procedures were conducted according to the standards described in our previous study (20). Specifically, a personalized ovarian stimulation protocol was developed based on patient age, body mass index (BMI), antral follicle count, and follicle-stimulating hormone (FSH) and anti-Müllerian hormone (AMH) levels. The ovarian stimulation protocols employed included short-acting, long-acting, antagonist, microstimulation, and natural cycles. When the diameter of the dominant follicle exceeded 18 mm, a dose of 5,000–10,000 IU of Human Chorionic Gonadotropin (HCG; Merck Serono, Switzerland) was administered to induce oocyte maturation. Oocyte retrieval was performed transvaginally under ultrasound guidance 36–38 h later. In the ICSI procedure, all mature oocytes were injected with a single sperm. During IVF, oocytes were fertilized with prepared sperm (at a concentration of 50,000–100,000 motile sperm/mL). Fertilization was assessed 16–18 h postinsemination, with normal fertilization defined by the presence of two distinct pronuclei. Subsequently, fertilized oocytes were placed in G1-plus and G2-plus (Vitrolife, Sweden) continuous culture media and incubated in a culture chamber at 37°C with 6% CO2 and 5% O2. At the blastocyst stage, morphological scoring was conducted following Gardner’s criteria to determine eligibility for biopsy (21). All blastocyst biopsies were performed using the hole-punch and laser-assisted cutting methods. The 4–6 trophoblast cells obtained from the biopsy were washed with PBS, placed in PCR tubes containing cell buffer, and stored at -20°C for chromosome testing. Blastocysts were cryopreserved within 1 h postbiopsy using a previously described method (22).

### 2.3 PGT program and result analysis

Biopsy samples were processed using the ChromSwift kit (Yikon, China) according to the manufacturer’s instructions, employing Multiple Annealing and Looping-Based Amplification Cycles for whole-genome amplification. Subsequently, the amplified products underwent library preparation, including adapter ligation, PCR amplification, and purification, resulting in a library size of 200–1,000 bp. The purified library was quantified using a Qubit instrument, and the ploidy status of the embryos was determined by sequencing on the Illumina MiSeq DX platform. Chromosome copy number variation (CNV) analysis was performed using the Chromgo software (Yikon, China), as previously described. An embryo was classified as euploid when the proportion of chromosomal aneuploidy was <30%. It was classified as aneuploid when the CNV size was ≥4 Mb and the proportion of chromosomal aneuploidy exceeded 70%. When the CNV size was ≥4 Mb and the proportion of chromosomal aneuploidy was between 30% and 70%, the embryo was classified as mosaic.

### 2.4 Embryo transfer and follow-up

All patients underwent frozen-thawed embryo transfer and single blastocyst transfer. Endometrial preparation protocols were developed based on specific patient characteristics, and they primarily hormone replacement therapy cycle, natural cycle, GnRH agonist cycle, and ovulation induction cycle. Prior to embryo transfer, transvaginal ultrasonography was performed to assess the uterus, with an endometrial thickness ≥8 mm considered suitable for embryo transfer. In the PGT-A group, the highest morphological grade euploid embryos were prioritized for thawing, whereas in the non-PGT-A group, the highest-scoring blastocysts were selected. The embryos were transferred into the uterine cavity 4–5 h after thawing.

Blood HCG levels were measured 14 days after embryo transfer. Clinical pregnancy was confirmed through ultrasonography at 4–6 weeks post-transfer and defined as the presence of a gestational sac containing an embryo with detectable cardiac activity. Fetal loss occurring before 20 weeks of gestation was classified as a miscarriage. Live birth was defined as the delivery of a newborn with vital signs at ≥24 weeks gestation. Preterm birth was defined as delivery of a viable fetus before 37 weeks gestation.

### 2.5 Statistical analysis

Data were analyzed using SPSS 27.0 (IBM Corporation, USA). Continuous variables with a normal distribution are expressed as mean ± standard deviation. Comparisons between two groups were performed using an independent samples t-test, whereas comparisons among three groups were conducted using one-way ANOVA. The variables analyzed included age, BMI, number of miscarriages, basal sex hormone levels, days of ovarian stimulation, total gonadotropin dose, number of retrieved oocytes, endometrial thickness, gestation days, birth length, and birth weight. For categorical variables expressed as percentages (%), intergroup comparisons were performed using the χ² test. When the theoretical frequency was less than 5, Fisher’s exact test was applied. Categorical variables included the blastocyst formation rate, embryo quality, biopsy day, endometrial preparation protocols, cause of infertility, and LBR/CPR ratio. We further conducted a multivariate logistic regression analysis on the clinical pregnancy rate, miscarriage rate, live birth rate, preterm birth rate, and sex ratio to adjust for the effects of potential confounding factors, such as maternal age, endometrium thickness, BMI, AMH, antral follicle count (AFC), embryo quality, endometrial preparation protocols, number of previous miscarriages. The results are expressed as adjusted odds ratios (OR) and 95% confidence intervals(CI). A P value < 0.05 was regarded as being statistically significant.

## 3 Results

### 3.1 Comparison of cycle baseline characteristics between the PGT-A and non-PGT-A groups in patients with RPL

This study analyzed 818 ovarian stimulation cycles in 797 patients with RPL, with 203 and 615 cycles in the PGT-A and non-PGT-A groups, respectively. **Table 1** presents a comparison of the baseline characteristics between the two groups. No significant differences were observed in female average age (33.98 ± 4.30 vs. 33.40 ± 4.22, P = 0.091), male average age (35.57 ± 4.93 vs. 35.50 ± 5.47, P = 0.882), BMI (22.39 ± 2.95 vs. 22.58 ± 3.93, P = 0.527), number of miscarriages (2.65 ± 0.73 vs. 2.50 ± 1.21, P = 0.116), endometrium thickness (9.15 ± 1.80 vs. 9.25 ± 1.95, P = 0.442) and hormone levels (FSH, LH, E2, P, AMH). The ovarian stimulation days, total gonadotropin dosage, average number of oocytes retrieved, and blastocyst formation rates (68.91% vs. 67.33%, P = 0.223), cause of infertility, endometrial preparation protocols, and quality of transplanted embryos were also similar between the two groups.

**Table 1:** Baseline characteristics of patients in the PGT-A group and non-PGT-A group among patients with RPL.

Characteristics	PGT-A group	non-PGT-A group	P
No. of couples (n)	200	597	–
No. of oocyte retrieval cycles (n)	203	615	–
Female age (years, average ± SD)	33.98±4.30	33.40±4.22	0.091
Male age (years, average ± SD)	35.57±4.93	35.50±5.47	0.882
Female BMI (kg/m^2^, average ± SD)	22.39±2.95	22.58±3.93	0.527
No. of miscarriages (average ± SD)	2.65±0.73	2.50±1.21	0.116
Cause of infertility			
*Female factor( %, n)	51.23%(104/203)	57.89%(356/615)	0.077
*Male factor( %, n)	17.24%(35/203)	18.54%(114/615)	
Combined factor ( %, n)	31.53%(64/203)	23.58%(145/615)	
Basal sexual hormone			
FSH (IU/L, average ± SD)	7.31±1.85	7.45±2.53	0.486
LH (IU/L, average ± SD)	4.49±1.75	4.75±2.22	0.136
E2 (pg/mL, average ± SD)	38.62±19.76	40.52±22.28	0.281
P (ng/mL, average ± SD)	0.98±2.39	0.72±1.45	0.059
AMH (ng/mL, average ± SD)	3.61±2.20	3.95±2.50	0.080
Gonadotropin stimulation days (average ± SD)	10.39±1.86	10.50±1.88	0.462
Total gonadotropin dose(average ± SD)	2603.10±781.80	2482.57±864.26	0.078
No. of retrieved oocytes (average ± SD)	12.82±6.40	13.70±6.92	0.110
blastocyst formation rate (%, n)	68.91%(1155/1676)	67.33%(3790/5629)	0.223
No. of embryo transfer cycles (n)	277	888	–
Endometrial preparation protocols			
Hormone replacement therapy cycle ( %, n)	38.99%(108/277)	37.61%(334/888)	0.084
Natural cycle( %, n)	45.85%(127/277)	40.43%(359/888)	
*GnRH agonist cycle( %, n)	5.05%(14/277)	8.33%(74/888)	
Ovulation induction cycle( %, n)	10.11%(28/277)	13.63%(121/888)	
Endometrium thickness(mm, average ± SD)	9.15±1.80	9.25±1.95	0.442
Quality of transplanted embryos			
*Good-quality( %, n)	86.28%(239/277)	82.55%(733/888)	0.144
*Fair and Poor-quality( %, n)	13.72%(38/277)	17.45%(155/888)	

*GnRH agonist cycle, Gonadotropin-releasing hormone agonist cycle.

*Female factor: tubal factor, ovulatory dysfunction, diminished ovarian reserve, endometriosis, uterine factor.

*Male factor: oligospermia, asthenospermia, teratospermia, azoospermia, sexual dysfunction.

*Good-quality indicates a blastocyst score of AA/AB/BA,Fair- quality indicates a blastocyst score of BB/AC, Poor-quality indicates a blastocyst score of CA/BC/CB.

### 3.2 Analysis of PGT-A results in patients with RPL

In the PGT-A group, 1,032 blastocysts were biopsied, of which 54.07% (558/1,032) were euploid, 33.33% (344/1,032) were aneuploid, and 12.60% (130/1,032) were mosaic. The proportion of high-quality embryos in the euploid blastocysts was significantly higher than that in the aneuploid and mosaic groups (85.66% vs. 70.35% vs. 78.46%, P < 0.01). Regarding developmental days, the proportion of Day 5 blastocysts was higher in the euploid group (44.98% vs. 31.69% vs. 23.85%, P < 0.01), whereas Day 6 and Day 7 blastocysts were more common in the aneuploid and mosaic groups (**Table 2**).

**Table 2:** PGT test results of blastocyst biopsy in patients with RPL.

Item	Euploid	Aneuploid	Mosaic	P
Number of biopsied embryos (%, n)	54.07%(558/1032)	33.33%(344/1032)	12.60%(130/1032)	-
Embryo quality				
*Good-quality, (%, n)	85.66%(478/558)	70.35%(242/344)	78.46%(102/130)	<0.01
*Fair and Poor-quality, (%, n)	14.34%(80/558)	29.65%(102/344)	21.54%(28/130)	
Biopsy day				
D5	44.98%(251/558)	31.69%(109/344)	23.85%(31/130)	<0.01
D6 and D7	55.02%(307/558)	68.31%(235/344)	76.15%(99/130)	

*Good-quality indicates a blastocyst score of AA/AB/BA,Fair- quality indicates a blastocyst score of BB/AC, Poor-quality indicates a blastocyst score of CA/BC/CB.

### 3.3 Age-stratified analysis of patients with RPL, comparing clinical and perinatal outcomes between the PGT-A and non-PGT-A groups

In the age-stratified analysis (**Table 3**), among patients aged <38 years, the PGT-A group had a significantly higher CPR (63.64% vs. 55.03%, OR 1.668, P = 0.004) and LBR (53.03% vs. 43.25%, OR 1.735, P = 0.002) than the non-PGT-A group. Among patients aged ≥38 years, the PGT-A group also had significantly higher CPR (50.63% vs. 35.42%, OR 1.970, P = 0.034) than the non-PGT-A group. The LBR (36.70% vs. 23.96%, P = 0.084) in the PGT-A group trended higher, although this difference was not statistically significant. No significant differences were observed between the two groups with respect to the miscarriage rate, preterm birth rate, gestational age, birth weight, or newborn sex. Within the PGT-A group, no significant differences were observed in pregnancy or perinatal outcomes between patients aged <38 years and those aged ≥38 years.

**Table 3:** A compares of the clinical outcomes and perinatal results of patients with RPL by age stratification between the PGT-A group and the non-PGT-A group.

Pregnancy outcomes	Aged <38 years				Aged ≥38 years					
	PGT-A group	non-PGT-A group	OR (95% CI)	P	PGT-A group	non-PGT-A group	OR (95% CI)	P	OR^a^ (95% CI)	P^a^
Number of transfer cycles (n)	198	696	–	–	79	192	–	–	–	–
Clinical pregnancy rate, % (n)	63.64% (126/198)	55.03% (383/696)	1.668 (1.175-2.368)	0.004	50.63% (40/79)	35.42% (68/192)	1.970 (1.051-3.692)	0.034	0.611 (0.335-1.115)	0.108
Miscarriage rate, % (n)	16.67% (21/126)	20.89% (80/383)	0.915 (0.540-1.549)	0.740	25% (10/40)	32.35% (22/68)	1.269 (0.506-3.185)	0.612	1.002 (0.414-2.425)	0.997
Live birth rate, % (n)	53.03% (105/198)	43.25% (301/696)	1.735 (1.234-2.439)	0.002	36.70% (29/79)	23.96% (46/192)	1.814 (0.923-3.564)	0.084	0.595 (0.321-1.101)	0.098
LBR/CPR, % (n)	83.33% (105/126)	78.59% (301/383)	–	0.250	72.50% (29/40)	67.65% (46/68)	–	0.193	–	–
Preterm birth rate, % (n)	14.29% (15/105)	11.30% (34/301)	1.423 (0.703-2.881)	0.327	6.90% (2/29)	13.04% (6/46)	0.595 (0.054-6.515)	0.671	0.397 (0.073-2.158)	0.285
Gestation days (average ± SD)	268.08 ± 12.03	269.19 ± 12.80	–	0.436	270.72 ± 11.65	265.52 ± 18.38	–	0.178	–	0.293
Birth Length (cm, average ± SD)	48.54 ± 2.57	48.81 ± 2.47	–	0.354	49.31 ± 1.58	48.89 ± 3.04	–	0.496	–	0.129
Birth weight (g, average ± SD)	3171.38 ± 564.79	3184.93 ± 567.96	–	0.833	3194.14 ± 381.33	3228.15 ± 622.89	–	0.792	–	0.801
Birth sex rate, male, % (n)	59.05% (62/105)	59.47% (179/301)	0.982 (0.614-1.571)	0.941	51.72% (15/29)	65.22% (30/46)	1.503 (0.445-5.079)	0.512	1.331 (0.507-3.491)	0.562
Birth sex rate, female, % (n)	40.95% (43/105)	40.53% (122/301)			48.28% (14/29)	34.78% (16/46)				

OR^a^ and P^a^ value refers to a comparison within the PGT group.

The OR (95% CI) and P value of clinical pregnancy rate, miscarriage rate, live birth rate, preterm birth rate, and birth sex rate were adjusted for the following confounders: endometrium thickness, BMI, AMH, AFC, embryo quality, endometrial preparation protocols, number of previous miscarriages.

### 3.4 Stratified analysis by miscarriage count in patients with RPL, comparing clinical and perinatal outcomes between the PGT-A and non-PGT-A groups

The stratified analysis by miscarriage count (**Table 4**) revealed that among patients with three miscarriages, the PGT-A group exhibited the most significant clinical advantages. The CPR was significantly higher in the PGT-A group than in the non-PGT-A group (74.70% vs. 42.13%, OR 3.902, P < 0.01), and the LBR was nearly twice the level (55.42% vs. 32.02%, OR 2.506, P = 0.001).

**Table 4:** Comparison of the clinical outcomes and perinatal results of patients with RPL by the number of previous miscarriage and stratified between the PGT-A group and non-PGT-A group.

Pregnancy outcomes	Two recurrent pregnancy losses				Three recurrent pregnancy losses				Four or more recurrent pregnancy losses				
	PGT-A group	non-PGT-A group	OR (95% CI)	P	PGT-A group	non-PGT-A group	OR (95% CI)	P	PGT-A group	non-PGT-A group	OR (95% CI)	P	P^a^
Number of transfer cycles (n)	138	642	–	–	83	178	–	–	56	68	–	–	–
Clinical pregnancy rate, % (n)	55.80% (77/138)	54.36% (349/642)	1.087 (0.729-1.620)	0.683	74.70% (62/83)	42.13% (75/178)	3.902 (2.146-7.097)	<0.01	48.21% (27/56)	39.71% (27/68)	1.757 (0.798-3.868)	0.161	0.006
Miscarriage rate, % (n)	9.09% (7/77)	22.06% (77/349)	0.404 (0.108-0.903)	0.027	24.19% (15/62)	22.67% (17/75)	2.149 (0.992-4.658)	0.052	33.33% (9/27)	29.63% (8/27)	2.098 (0.601-7.328)	0.245	0.019
Live birth rate, % (n)	50.52% (70/138)	42.21% (271/642)	1.453 (0.979-2.156)	0.064	55.42% (46/83)	32.02% (57/178)	2.506 (1.432-4.384)	0.001	32.14% (18/56)	27.94% (19/68)	1.913 (0.755-4.846)	0.171	0.245
LBR/CPR, % (n)	90.91% (70/77)	77.65% (271/349)	–	0.008	74.19% (46/62)	76.00% (57/75)	–	0.808	66.67% (18/27)	70.37% (19/27)	–	0.770	–
Preterm birth rate, % (n)	10.00% (7/70)	10.66% (29/272)	1.098 (0.445-2.706)	0.839	10.87% (5/46)	12.28% (7/57)	1.573 (0.369-6.697)	0.540	27.78% (5/18)	21.05% (4/19)	2.085 (0.541-8.051)	0.122	0.543
Gestation days (average ± SD)	269.97 ± 9.87	269.13 ± 13.11	–	0.616	267.48 ± 13.91	268.34 ± 13.15	–	0.751	266.50 ± 13.95	263.79 ± 21.36	–	0.652	0.939
Birth Length (cm, average ± SD)	48.86 ± 2.03	48.84 ± 2.50	–	0.962	48.28 ± 2.80	48.84 ± 2.52	–	0.285	49.22 ± 2.62	48.37 ± 3.35	–	0.396	0.284
Birth weight (g, average ± SD)	3210.07 ± 427.96	3180.98 ± 574.46	–	0.692	3100.76 ± 624.54	3294.40 ± 543.19	–	0.094	3238.06 ± 625.76	3011.05 ± 647.94	–	0.286	0.484
Birth sex rate, male, % (n)	61.43% (43/70)	61.99% (168/271)	1.040 (0.601-1.801)	0.888	50.00% (23/46)	50.88% (29/57)	1.037 (0.450-2.388)	0.932	61.11% (11/18)	63.16% (12/19)	0.283 (0.042-1.935)	0.198	0.338
Birth sex rate, female, % (n)	38.57% (27/70)	38.01% (103/271)			50.00% (23/46)	49.12% (28/57)			38.89% (7/18)	36.84% (7/19)			

P^a^ refers to a comparison within the PGT group.

The OR (95% CI) and P value of the clinical pregnancy rate, miscarriage rate, live birth rate, preterm birth rate, birth sex rate were adjusted for the following confounders: maternal age, endometrium thickness, BMI, AMH, AFC, embryo quality, endometrial preparation protocols, number of previous miscarriages.

Among patients with two miscarriages, no significant difference in the CPR was observed between the two groups (55.80% vs. 54.36%, P = 0.683). Although the LBR was higher in the PGT-A group than in the non-PGT-A group (50.52% vs. 42.21%, P = 0.064), the difference was not statistically significant. Furthermore, the miscarriage rate was significantly lower in the PGT-A group than in the non-PGT-A group (9.09% vs. 22.06%, OR 0.404, P = 0.027). More importantly, the LBR/CPR ratio in the PGT-A group was significantly higher than that in the non-PGT-A group (90.91% vs. 77.65%, P = 0.008).

In patients with four or more miscarriages, no significant differences in the CPR (48.21% vs. 39.71%, P = 0.161) and LBR (32.14% vs. 27.94%, P = 0.171), and miscarriage rates (33.33% vs. 29.63%, P = 0.245) were observed between the PGT-A and non-PGT-A groups. Regarding perinatal outcomes, no significant differences were observed in gestation days, newborn length, birth weight, and sex ratio (P > 0.05) across all subgroups stratified by miscarriage count (two, three, or four or more). Within the PGT-A group, patients with three miscarriages had significantly higher CPR than the other groups (P = 0.006), while those with two miscarriages had a significantly lower miscarriage rate than the other groups (P = 0.019).

### 3.5 Stratified analysis of chromosomal polymorphism in patients with RPL, comparing the clinical and perinatal outcomes between the PGT-A and non-PGT-A groups

The stratified analysis by chromosomal polymorphisms (**Table 5**) showed that among patients without polymorphisms, the CPR (61.76% vs. 51.95%, OR 1.762, P < 0.001) and LBR (50.00% vs. 39.54%, OR 1.820, P < 0.01) were significantly higher in the PGT-A group than in the non-PGT-A group. Among those with polymorphisms, no significant difference was observed in clinical outcomes between the two groups. Regarding perinatal outcomes, no significant differences were observed between PGT-A group and non-PGT-A group, regardless of chromosomal polymorphism status. Within the PGT-A group, no significant differences were observed in pregnancy or perinatal outcomes between patients with normal chromosomes and chromosomal polymorphism.

**Table 5:** Comparison of clinical outcomes and perinatal results of patients with RPL by chromosomal polymorphisms and stratified between the PGT-A group and non-PGT-A group.

Pregnancy outcomes	Normal chromosomes				Chromosomal polymorphism					
	PGT-A group	non-PGT-A group	OR (95% CI)	P	PGT-A group	non-PGT-A group	OR (95% CI)	P	OR^a^ (95% CI)	P^a^
Number of transfer cycles (n)	238	822	–	–	39	66	–	–	–	–
Clinical pregnancy rate, % (n)	61.76% (147/238)	51.95% (427/822)	1.762 (1.273-2.440)	<0.001	48.72% (19/39)	36.36% (24/66)	2.421 (0.893-6.560)	0.082	0.583 (0.283-1.202)	0.144
Miscarriage rate, % (n)	19.05% (28/147)	23.42% (100/427)	0.950 (0.595-1.517)	0.830	15.79% (3/19)	8.33% (2/24)	2.298 (0.244-21.686)	0.467	0.635 (0.177-2.277)	0.486
Live birth rate, % (n)	50.00% (119/238)	39.54% (325/822)	1.820 (1.322-2.507)	<0.01	38.46% (15/39)	33.33% (22/66)	1.886 (0.653-5.443)	0.241	0.6400.308-1.332)	0.233
LBR/CPR, % (n)	80.95% (119/147)	76.11% (325/427)	–	0.227	78.95% (15/19)	91.67% (22/24)	–	0.380	–	–
Preterm birth rate, % (n)	31.09% (37/119)	4.91% (16/326)	1.354 (0.685-2.677)	0.384	6.67% (1/15)	13.64% (3/22)	–	0.992	0.361 (0.042-3.106)	0.353
Gestation days (average ± SD)	268.40 ± 12.36	268.87 ± 13.42	–	0.743	270.60 ± 8.13	269.32 ± 10.49	–	0.693	0.505	0.505
Birth Length (cm, average ± SD)	48.66 ± 2.48	48.84 ± 2.56	–	0.502	270.60 ± 8.13	269.32 ± 10.49	–	0.693	0.471	0.471
Birth weight (g, average ± SD)	3161.18 ± 529.24	3193.09 ± 581.69	–	0.600	49.13 ± 1.73	48.50 ± 2.44	–	0.939	0.353	0.353
Birth sex rate, male, % (n)	57.98% (69/119)	59.20% (193/326)	1.004 (0.644-1.567)	0.985	53.33% (8/15)	72.72% (16/22)	2.467 (0.304-18.791)	0.383	1.292 (0.401-4.162)	0.668
Birth sex rate, female, % (n)	42.02% (50/119)	40.49% (132/326)			46.67% (7/15)	27.27% (6/22)				

OR^a^ (odds ratio) and P^a^ value refers to the comparison within the PGT group.

The OR (95% CI) and P value of clinical pregnancy rate, miscarriage rate, live birth rate, preterm birth rate, birth sex rate were adjusted for the following confounders: maternal age, endometrium thickness, BMI, AMH, AFC, embryo quality, endometrial preparation protocols, number of previous miscarriages.

### 3.6 Stratified analysis of ovarian reserve in patients with RPL, comparing clinical and perinatal outcomes between the PGT-A and non-PGT-A groups

Stratified analysis by ovarian reserve revealed that among patients with normal ovarian reserve, the PGT-A group had significantly higher CPR (59.68% vs. 52.37%, OR 1.584, P = 0.004) and LBR (49.41% vs. 40.56%, OR 1.720, P < 0.01) than the non-PGT-A group (**Table 6**). In patients with diminished ovarian reserve, the PGT-A group maintained a higher CPR (62.50% vs. 39.45%, OR 4.848, P = 0.015). However, due to a significantly higher miscarriage rate compared to the non-PGT-A group (40.00% vs. 23.36%, OR 2.274, P = 0.029), the improvement in LBR was not significant (37.50% vs. 28.44%, P = 0.257). No significant differences in perinatal outcomes were observed between the PGT-A group and non-PGT-A group, regardless of ovarian reserve function. Within the PGT-A group, no significant differences were observed in pregnancy or perinatal outcomes between patients with normal ovarian reserve and diminished ovarian reserve.

**Table 6:** Comparison of clinical outcomes and perinatal results for patients with RPL by ovarian reserve function and stratified between the PGT-A group and non-PGT-A group.

Pregnancy outcomes	Normal ovarian reserve				Diminished ovarian reserve				PGT group	
	PGT-A group	non-PGT-A group	OR (95% CI)	P	PGT-A group	non-PGT-A group	OR (95% CI)	P	OR (95% CI)	P
Number of transfer cycles(n)	253	779	–	–	24	109	–	–	–	–
Clinical pregnancy rate, % (n)	59.68% (151/253)	52.37% (408/779)	1.584 (1.156-2.171)	0.004	62.50% (15/24)	39.45% (43/109)	4.848 (1.363-7.247)	0.015	1.428 (0.521-3.912)	0.488
Miscarriage rate, % (n)	16.56% (25/151)	22.55% (92/408)	0.796 (0.487-1.299)	0.361	40.00% (6/15)	23.36% (10/43)	2.274 (1.181-3.553)	0.029	2.205 (0.649-7.490)	0.205
Live birth rate, % (n)	49.41% (125/253)	40.56% (316/779)	1.720 (1.258-2.352)	<0.01	37.50% (9/24)	28.44% (31/109)	2.103 (0.600-6.759)	0.257	0.843 (0.307-2.312)	0.739
LBR/CPR, %(n)	82.78% (125/151)	77.45% (316/408)	–	0.961	60.00% (9/15)	72.09% (31/43)	–	0.383	–	–
Preterm birth rate, % (n)	13.60% (17/125)	12.03% (38/316)	1.147 (0.587-2.241)	0.688	22.22% (2/9)	0	–	–	–	–
Gestation days (average ± SD)	268.42±12.29	268.64±13.51	–	0.871	271.89±4.57	269.30±15.51	–	0.626	–	0.984
Birth Length (cm, average ± SD)	48.70±2.45	48.78±2.54	–	0.779	48.78±1.72	49.18±2.65	–	0.669	–	0.930
Birth weight(g, average ± SD)	3169.36±535.36	3194.62±544.68	–	0.659	3272.78±451.10	3152.36±818.96	–	0.676	–	0.573
Birth sex rate, male , % (n)	56.00% (70/125)	60.76% (192/316)	1.165 (0.747-1.817)	0.502	77.78% (7/9)	54.84%(17/31)	0.271 (0.031-2.400)	0.241	0.450 (0.075-2.697)	0.382
Birth sex rate, female , % (n)	44.00% (55/125)	39.24% (124/316)			22.22% (2/9))	45.16%(14/31)				

OR^a^ (odds ratio) and P^a^ value refers to the comparison within the PGT group.

The OR (95% CI) and P value of clinical pregnancy rate, miscarriage rate,mive birth rate, mreterm birth rate, birth sex rate were adjusted for the following confounders: maternal age 、endometrium thickness, BMI, AMH, AFC, embryo quality、embryo transfer protocol、number of previous miscarriages.

## 4 Discussion

This study was a retrospective analysis evaluating the clinical efficacy of PGT-A in patients with RPL subgroups, including 818 oocyte retrieval cycles and 1,165 single blastocyst transfer cycles. PGT-A was associated with significant improvements in clinical pregnancy and live birth rates in patients with RPL, particularly among those with a maternal age < 38 years, those with a history of three miscarriages, patients with normal chromosomal karyotypes, and those with normal ovarian reserve. These findings provide important evidence for the rational application of PGT-A in the management of RPL. Notably, no significant differences were observed in perinatal outcomes between the PGT-A and non-PGT-A groups in this study, aligning with findings from previous reports (23–25).

In a cohort study of PGT-A in patients with RPL, 1,032 blastocysts were biopsied, with euploid embryos accounting for 54.07%. This result is consistent with previous findings in similar populations (26, 27). Euploid embryos exhibited a superior morphological quality (with a high-quality embryo rate of 85.66%) and more favorable developmental dynamics (with a Day 5 blastocyst rate of 44.98%) compared to aneuploid embryos, further confirming the association between chromosomal status and embryonic developmental potential (20, 28).

This study found that PGT-A was associated with improved pregnancy outcomes in patients with RPL, though its efficacy varied by maternal age. Among women aged ≥38 years, PGT-A led to a statistically significant increase in the clinical pregnancy rate (50.63% vs. 35.42%, OR 1.970, P = 0.034) and a clinically meaningful, although not statistically significant, improvement in live birth rate (36.70% vs. 23.96%, P = 0.084). Although some studies supports the use of PGT-A in older women due to the higher risk of aneuploid embryos (4, 26, 29–31), our findings are consistent with the results of the *post hoc* subgroup analysis of the STAR trial, which also observed a significant increase in the ongoing pregnancy rate per transfer cycle when using PGT-A in women aged 35–40 years (29). However, a critical issue was raised by Pagliardini et al. (32) in their reanalysis of the STAR data: the biopsy process may lead to the wastage of embryos with developmental potential due to misdiagnosis or embryo damage, with an embryo loss rate as high as 39.2% (33). This provides an important perspective for explaining the phenomenon observed in our study, where the live birth rate in older patients remains limited even when euploid embryos are selected through PGT-A. Therefore, the potential harm from biopsy may partially offset the benefits of aneuploidy screening.

Our study reveals the boundaries of the effectiveness of PGT-A technology and the complex physiological background of advanced maternal age fertility. In this respect, our data indicate that PGT-A can effectively address the issue of implantation failure caused by chromosomal abnormalities by selecting euploid embryos, thereby significantly improving clinical pregnancy rates. However, once a euploid embryo successfully implants, the ability to maintain the pregnancy to live birth may depend more on other factors closely related to advanced age that PGT-A technology cannot intervene in. These factors may include: (a) the intrinsic non-chromosomal developmental potential of the embryo: euploid embryos may still harbor other genetic or epigenetic defects, the risk of which increases with parental age; (b) systemic aging of the maternal intrauterine environment: changes *in utero*placental blood flow perfusion and the immune microenvironment may affect the continued growth and development of the fetus during mid to late pregnancy (34);(c) age-related differences in the “embryonic self-correction” ability may be a key factor involved. Research by Victor et al. (35) suggests that embryos from younger patients may possess a more robust intrinsic capacity to correct or compensate for their chromosomal abnormalities (for instance, through selective apoptosis or the proliferation of dominant cell lines), whereas this “self-correction” potential may diminish in embryos derived from older sources. Even when screened as euploid through PGT-A, the developmental potential of embryos from older sources may be compromised due to oocyte aging itself. This age-related decline in intrinsic potential limits the ongoing developmental capacity of embryos post-implantation, a limitation that PGT-A cannot overcome.

For younger patients with RPL (< 38 years), previous studies have reported inconsistent findings regarding PGT-A efficacy. Our study found that the clinical pregnancy rate and live birth rate in the PGT-A group of younger patients increased by 8.61% (63.64% vs. 55.03%, OR 1.668, P = 0.004) and 9.78% (53.03% vs. 43.25%, OR 1.735, P = 0.002), respectively, compared to the non-PGT-A group. This finding is consistent with those of previous studies (36). However, Xiao and Yan et al. reported no significant differences (26, 37). In addition, Liu et al. reported that young patients with RPL may continue to experience high miscarriage rates even with PGT-A (6); however, this phenomenon was not observed in our study, and this discrepancy may stem from the following reasons: 1) the average patient age in our cohort was comparatively younger(33.98 ± 4.30 years); 2) we used more stringent embryonic morphological assessment criteria; and 3) we employed optimized laboratory culture conditions. The STAR trial found that, among reproductive-age women with a good prognosis (ages 25-40), PGT-A did not improve live birth rates compared to morphological selection in an intention-to-treat analysis (29). A subsequent meta-analysis by Taskin et al. (38) further confirmed that PGT-A failed to significantly enhance ongoing pregnancy rates or clinical pregnancy rates in a general population that included various age groups. However, these studies primarily included a ‘good prognosis’ general IVF population rather than specifically targeting RPL patients. The RPL patient group is unique, as embryonic aneuploidy is considered one of the main causes of early pregnancy loss. This study focuses on this specific population and through a detailed stratified analysis finds that younger RPL patients (< 38 years) significantly benefit from PGT-A. This finding does not contradict the negative conclusions drawn from studies in non-selective or general populations, but rather highlights the importance of patient selection in assessing the value of PGT-A. Furthermore, the pregnancy outcomes for RPL patients under 38 years of age within the PGT-A group were superior to those aged 38 and above (clinical pregnancy rate: 63.64% vs. 50.63%, P = 0.046; live birth rate: 53.03% vs. 36.70%, P = 0.014), further confirming the impact of age on reproductive potential (39).

The number of previous natural miscarriages may be an important prognostic factor influencing subsequent pregnancy outcomes. This study presents clinically significant findings based on a stratified analysis by miscarriage frequency. The analysis revealed that PGT-A was most effective in patients who had experienced three prior miscarriages, with clinical pregnancy and live birth rates rising to 74.70% and 55.42%, respectively; these results were significantly higher than those in the non-PGT-A group (42.13% and 32.02%, respectively). For patients with two prior miscarriages, PGT-A did not significantly improve clinical pregnancy and live birth rates, but it was associated with a significant reduction in miscarriage rates (9.09% vs. 22.06%, OR 0.404, P = 0.027). This finding is partially consistent with that of Wang et al., who suggested that a patient’s history of miscarriage is not associated with HCG positivity, ongoing pregnancies, or total pregnancy loss outcomes (18, 40). More importantly, the LBR/CPR ratio in the PGT-A group was significantly higher than that in the non-PGT-A group (90.91% vs. 77.65%, P = 0.008). This indicates that the primary advantage of PGT-A for this subgroup of patients lies in improving the efficiency of live birth conversion after clinical pregnancy, significantly reducing the risk of pregnancy loss after implantation. Notably, our research indicates that the effectiveness of PGT-A significantly diminishes when ≥ 4 spontaneous abortions have occurred (clinical pregnancy rate: 48.21% vs. 39.71%, OR 1.757, P = 0.161; live birth rate: 32.14% vs. 27.94%, OR 1.913, P = 0.171). This finding suggests that as the number of miscarriages increases, mechanisms beyond chromosomal abnormalities in embryos (such as decreased endometrial receptivity and immune-coagulation abnormalities) gradually become the dominant factors contributing to pregnancy failure (41). Therefore, for such patients, clinical decision-making should not rely solely on PGT-A but should also include simultaneous investigation and intervention of endometrial and immune factors to improve the overall chances of live birth. Given that the sample size of this subgroup is relatively small, its clinical significance needs to be further confirmed by studies with larger samples.

However, the effect of chromosomal polymorphisms on the outcomes of PGT-A remains unclear. This study provides an in-depth analysis of chromosomal polymorphisms that are often overlooked. The results indicated that in patients without polymorphisms, the clinical pregnancy and live birth rates in the PGT-A group were significantly higher than those in the non-PGT-A group (61.76% vs. 51.95%, OR 1.762, P<0.001; 50.00% vs. 39.54%, OR 1.820, P<0.01, respectively). However, in patients with polymorphisms, the differences between the two groups were not statistically significant. This finding suggests that PGT-A may be more suitable for patients with RPL who have a normal chromosomal karyotype and may not fully address the reproductive challenges posed by certain chromosomal polymorphic variations. This aligns with the conclusions of the systematic review by Iews et al. (38), which found that for couples experiencing RPL due to chromosomal structural rearrangements (such as reciprocal translocations and Robertsonian translocations), PGT does not demonstrate a clear advantage in live birth rates compared to natural conception, while significantly increasing costs. Our study extends this cautious perspective to populations with chromosomal polymorphisms, suggesting that for patients with such complex genetic backgrounds, PGT-A may not be the preferred or sole solution. Although the sample size of patients with polymorphisms was small (39 cases in the PGT-A group), the clinical pregnancy rate in the PGT-A group was higher than that in the non-PGT-A group (48.72% vs. 36.36%, OR 2.421, P = 0.082), indicating that some patients with polymorphisms may still benefit from PGT-A. Future studies should involve a larger sample size and integrate epigenetic and endometrial function assessments to clarify the optimal treatment strategies for patients with chromosomal polymorphisms.

Ovarian reserve is a core indicator of reproductive potential. This study found that in patients with normal ovarian reserve, PGT-A significantly increased the clinical pregnancy (59.68% vs 52.37%, OR 1.584, P = 0.004) and live birth rates (49.41% vs 40.56%, OR 1.720, P<0.01). This suggests that selecting euploid embryos can effectively improve transplantation efficiency and reduce ineffective transfers, a conclusion consistent with the findings of Mumusoglu et al. (42). They proposed that PGT-A is beneficial for patients with sufficient ovarian reserve, as it can shorten the time to live birth, and further confirmed that when the number of oocytes is adequate, embryo aneuploidy remains the primary barrier to achieving live birth. However, we observed a markedly different outcome in patients with diminished ovarian reserve. Although PGT-A improved the CPR (62.50% vs. 39.45%, OR 4.848, P = 0.015), its benefits appeared to be limited. Analysis of the LBR/CPR ratio indicated a trend towards decreased pregnancy maintenance efficiency in the PGT-A group (60.00% vs. 72.09, p=0.383). This is because the increase in CPR was significantly offset by a higher miscarriage rate (40.00% vs. 23.36%, OR 2.274, P = 0.029), ultimately failing to translate into a significant improvement in live birth rate (37.50% vs. 28.44%, OR 2.103, P = 0.257).

This suggests that while PGT-A may effectively select embryos with implantation potential for the DOR population, these embryos, generated in the context of advanced maternal age or diminished ovarian function, may possess intrinsic developmental potential defects beyond chromosomal abnormalities, leading to difficulties in sustaining development to live birth. This indicates that in patients with RPL, even those with low ovarian reserve who possess euploid embryos may still experience pregnancy loss because of factors such as impaired endometrial receptivity or insufficient luteal function. This finding is consistent with the results of Sun et al. (43), who analyzed 1,982 cycles of frozen-thawed single euploid embryo transfer confirmed by PGT-A and revealed a significantly increased miscarriage risk in patients with low ovarian reserve. These findings further suggest that the elevated miscarriage rate in this population is not due to increased embryonic aneuploidy; rather, it stems from the compromised endometrial decidualization and luteal function, which lead to post implantation failure of euploid embryos.

This study had a few limitations. First, the retrospective design may introduce selection bias. Second, some subgroup sample sizes are small (e.g., patients with polymorphisms), potentially limiting statistical power. Lastly, other potential influencing factors, including endometrial receptivity and immune factors, were not evaluated.

In conclusion, this study suggests that the application of PGT-A is associated with improved pregnancy outcomes in the subgroup of RPL patients, but its benefits exhibit clear demographic differences and boundaries of effect. In patients younger than 38 years, with a history of three miscarriages, normal chromosomal karyotype, and normal ovarian reserve, the use of PGT-A is significantly correlated with increased clinical pregnancy rates and live birth rates. For patients with a history of two miscarriages, although PGT-A did not significantly increase clinical pregnancy rates, it significantly reduced the risk of miscarriage and improved the conversion efficiency from clinical pregnancy to live birth. However, in patients with diminished ovarian reserve, while PGT-A is associated with an increase in clinical pregnancy rates, this benefit is offset by the subsequent increase in miscarriage risk, failing to translate into an improvement in live birth rates. Additionally, in patients with chromosomal polymorphisms or a history of four or more miscarriages, this study did not observe significant clinical benefits from PGT-A. For these populations, a cautious and personalized approach is recommended when considering PGT-A. However, given the limited sample size of these subgroups, the related conclusions require further validation through larger-scale studies. This study emphasizes the importance of implementing precise classification and individualized treatment strategies in the clinical management of RPL, providing important evidence for identifying advantageous populations that may benefit from PGT-A, which aids in the optimization of RPL management.

## Data Availability

The data that support the findings of this study have beendeposited into CNGB Sequence Archive (CNSA) of China NationalGeneBank DataBase (CNGBdb) with accession number CNP0008478.
